# Supplementary material for: Novel evidence that elk were historically native to the Sierra Nevada, and recent range expansions into the region
Source: PLoS One. 2024 Sep 18;19(9):e0301665. doi: 10.1371/journal.pone.0301665 (PMC11410259; doi:10.1371/journal.pone.0301665)

PLOS ONE

Novel evidence that elk were historically native to the Sierra Nevada, and recent range expansions into the region

--Manuscript Draft--

**Manuscript Number:**	PONE-D-23-41818
**Article Type:**	Research Article
**Full Title:**	Novel evidence that elk were historically native to the Sierra Nevada, and recent range expansions into the region
**Short Title:**	Evidence of elk in the Sierra Nevada
**Corresponding Author:**	Richard B. Lanman, MD<br>Institute for Historical Ecology, North Santa Clara Resource Conservation District<br>Los Altos, CA UNITED STATES
**Keywords:**	Cervus canadensis, Cervus elaphus, elk, historic range, historical ecology, Sierra Nevada
**Abstract:**	Elk (Cervus canadensis) have not been thought to be historically native to the Sierra Nevada mountain range of California and Nevada. However, the Rocky Mountain elk subspecies has steadily increased its range southward into the northern Sierra Nevada over the last century. Recent reports also reveal Rocky Mountain elk moving northwards into the southern Sierra Nevada. Dispersal of lone bull elk from 2019-2022 have occurred to the central Sierra Nevada south of Lake Tahoe. Recent range expansions of elk herds and long-distance dispersals of individual elk raised questions about the possible historical presence of elk throughout this mountain range. Herein we conducted a broad investigation into historical newspaper accounts and other early explorer and naturalist observer records, museum specimens, late Holocene zooarchaeological records, and indirect evidence including toponomastic references and Native American ethnographic and ethnolinguistic information. Taken in total, a variety of data sources suggest elk once inhabited portions of the Sierra Nevada. Positive records were not numerous, suggesting that historically elk were scarce, or alternatively, suppressed by aboriginal hunting, or nearly extirpated by Anglo-Americans in the California Fur Rush in the early nineteenth century.
**Order of Authors:**	Richard B. Lanman, MD
	Thomas J. Batter, PhD
	Cody J. McKee, MS
**Additional Information:**	
**Question**	**Response**
**Financial Disclosure**<br><br>Enter a financial disclosure statement that describes the sources of funding for the work included in this submission. Review the submission guidelines for detailed requirements. View published research articles from *PLOS ONE* for specific examples.<br><br>This statement is required for submission and **will appear in the published article** if the submission is accepted. Please make sure it is accurate.	The author(s) received no specific funding for this work.

Powered by Editorial Manager® and ProduXion Manager® from Aries Systems Corporation

**Funded studies**<br>Enter a statement with the following details:<br>• Initials of the authors who received each award<br>• Grant numbers awarded to each author<br>• The full name of each funder<br>• URL of each funder website<br>• Did the sponsors or funders play any role in the study design, data collection and analysis, decision to publish, or preparation of the manuscript?<br><br>Did you receive funding for this work?	
**Competing Interests**<br><br>Use the instructions below to enter a competing interest statement for this submission. On behalf of all authors, disclose any competing interests that could be perceived to bias this work—acknowledging all financial support and any other relevant financial or non-financial competing interests.<br><br>This statement is required for submission and **will appear in the published article** if the submission is accepted. Please make sure it is accurate and that any funding sources listed in your Funding Information later in the submission form are also declared in your Financial Disclosure statement.<br><br>View published research articles from *PLOS ONE* for specific examples.	NO authors have competing interests

**NO authors have competing interests**<br><br>Enter: *The authors have declared that no competing interests exist.*<br><br>**Authors with competing interests**<br><br>Enter competing interest details beginning with this statement:<br><br>*I have read the journal's policy and the authors of this manuscript have the following competing interests: [insert competing interests here]*<br><br>* typeset	
**Ethics Statement**<br><br>Enter an ethics statement for this submission. This statement is required if the study involved:<br><br>• Human participants<br>• Human specimens or tissue<br>• Vertebrate animals or cephalopods<br>• Vertebrate embryos or tissues<br>• Field research<br><br>Write "N/A" if the submission does not require an ethics statement.<br><br>General guidance is provided below. Consult the submission guidelines for detailed instructions. **Make sure that all information entered here is included in the Methods section of the manuscript.**	N/A

Powered by Editorial Manager® and ProduXion Manager® from Aries Systems Corporation

**Format for specific study types**<br><br>**Human Subject Research (involving human participants and/or tissue)**<br>• Give the name of the institutional review board or ethics committee that approved the study<br>• Include the approval number and/or a statement indicating approval of this research<br>• Indicate the form of consent obtained (written/oral) or the reason that consent was not obtained (e.g. the data were analyzed anonymously)<br><br>**Animal Research (involving vertebrate animals, embryos or tissues)**<br>• Provide the name of the Institutional Animal Care and Use Committee (IACUC) or other relevant ethics board that reviewed the study protocol, and indicate whether they approved this research or granted a formal waiver of ethical approval<br>• Include an approval number if one was obtained<br>• If the study involved *non-human primates*, add *additional details* about animal welfare and steps taken to ameliorate suffering<br>• If anesthesia, euthanasia, or any kind of animal sacrifice is part of the study, include briefly which substances and/or methods were applied<br><br>**Field Research**<br><br>Include the following details if this study involves the collection of plant, animal, or other materials from a natural setting:<br>• Field permit number<br>• Name of the institution or relevant body that granted permission	
**Data Availability**<br><br>Authors are required to make all data underlying the findings described fully available, without restriction, and from the time of publication. PLOS allows rare exceptions to address legal and ethical concerns. See the PLOS Data Policy and FAQ for detailed information.	Yes - all data are fully available without restriction

A Data Availability Statement describing where the data can be found is required at submission. Your answers to this question constitute the Data Availability Statement and **will be published in the article**, if accepted.<br><br>**Important:** Stating 'data available on request from the author' is not sufficient. If your data are only available upon request, select 'No' for the first question and explain your exceptional situation in the text box.<br><br>Do the authors confirm that all data underlying the findings described in their manuscript are fully available without restriction?	
**Describe where the data may be found in full sentences. If you are copying our sample text, replace any instances of XXX with the appropriate details.**<br><br>• If the data are **held or will be held in a public repository**, include URLs, accession numbers or DOIs. If this information will only be available after acceptance, indicate this by ticking the box below. For example: *All XXX files are available from the XXX database (accession number(s) XXX, XXX.*).<br>• If the data are all contained **within the manuscript and/or Supporting Information files**, enter the following: *All relevant data are within the manuscript and its Supporting Information files.*<br>• If neither of these applies but you are able to provide **details of access elsewhere**, with or without limitations, please do so. For example:<br><br>*Data cannot be shared publicly because of [XXX]. Data are available from the XXX Institutional Data Access / Ethics Committee (contact via XXX) for researchers who meet the criteria for access to confidential data.*<br><br>*The data underlying the results presented in the study are available from (include the name of the third party*	All relevant data are within the manuscript and its Supporting Information files.

and contact information or URL).<br>• This text is appropriate if the data are owned by a third party and authors do not have permission to share the data.<br><br>* typeset	
Additional data availability information:	

Manuscript

Click here to access/download;Manuscript;Elk in the Sierra Nevada FINAL 2023.12.12.docx

Novel evidence that elk were historically native to the Sierra Nevada, and recent range expansions into the region

Short title: Evidence of elk in the Sierra Nevada

Authors: RICHARD B. LANMAN[1¶*], THOMAS J. BATTER[2¶], CODY J. MCKEE[3¶]

[1] Institute for Historical Ecology, Los Altos, California, USA

[2] Elk and Pronghorn Program, Big Game Unit, Wildlife Branch, California Department of Fish and Wildlife, West Sacramento, California, USA

[3] Elk and Moose Program, Game Division, Nevada Department of Wildlife, Reno, Nevada, USA

* Corresponding Author: Richard B. Lanman

Email: ricklanman@gmail.com (RBL)

[¶] These authors contributed equally to this work.

Key words: *Cervus canadensis*, *Cervus elaphus*, elk, historical range, historical ecology, Sierra Nevada

Abstract

Elk (Cervus canadensis) have not been thought to be historically native to the Sierra Nevada mountain range of California and Nevada. However, the Rocky Mountain elk subspecies

has steadily increased its range southward into the northern Sierra Nevada over the last century. Recent reports also reveal Rocky Mountain elk moving northwards into the southern Sierra Nevada. Dispersal of lone bull elk from 2019-2022 have occurred to the central Sierra Nevada south of Lake Tahoe. Recent range expansions of elk herds and long distance dispersals of individual elk raised questions about the possible historical presence of elk throughout this mountain range. Herein we conducted a broad investigation into historical newspaper accounts and other early explorer and naturalist observer records, museum specimens, late Holocene zooarchaeological records, and indirect evidence including toponomastic references and Native American ethnographic and ethnolinguistic information. Taken in total, a variety of data sources suggest elk once inhabited portions of the Sierra Nevada. Positive records were not numerous, suggesting that historically elk were scarce, or alternatively, suppressed by aboriginal hunting, or nearly extirpated by Anglo-Americans in the California Fur Rush in the early nineteenth century.

Introduction

The California Department of Fish and Wildlife (CDFW) 2018 Elk Conservation and Management Plan depicted historical elk (*Cervus canadensis*) distribution as being absent from the Sierra Nevada of eastern California and western Nevada (Fig 1) (1). This estimate was based on twentieth century accounts written after possible extirpation of elk in the nineteenth century in the region (2,3). The nearest elk historically were thought to be Rocky Mountain elk (*C. c. nelsoni*) located in northeast California's southern Cascade Range and tule elk (*C. c. nannodes*) relegated to "the Central Valley and the grasslands and woodlands of central California's Coast Range" (Fig. 1). Tule elk have a well-established habitat preference for more open terrain and generally avoid forested habitat and higher elevations characteristic of the Sierra Nevada (3).

This strong habitat preference is likely related to physical and behavioral adaptations for drier, Mediterranean-climate conditions (4,5). In describing primitive North American elk distribution in 1951, O.J. Murie cited a lack of available records of elk in easternmost and southernmost California and suggested that elk did not occupy Nevada “in any considerable numbers”. Murie recognized, however, that elk have occurred beyond the boundaries presented and a more extensive record would likely reveal a greater historical distribution (2). In 1969 D.R. McCullough identified reliable historical records and museum specimens consistent with Rocky Mountain elk historical presence in Shasta and Siskiyou Counties of northeastern California (3,6,7). Until then California Fish and Game had disputed that this subspecies had existed in California, aligning with Murie’s perspective (1,8). McCullough’s findings were accepted by CDFW in their 2018 elk plan (1).

Fig 1. Estimated historical distribution of California’s three elk subspecies (reproduced from CDFW Elk Conservation and Management Plan)**.** Rocky Mountain elk (yellow) shown in northeastern California north of the Sierra Nevada, Roosevelt elk (blue) in northwestern California, and tule elk (pink) in California’s Central Valley and central and central coast regions. As shown, elk were not recognized as historically present in the Sierra Nevada.

In partial agreement with Murie’s work, the 1997 Nevada Department of Wildlife (NDOW) Elk Species Management Plan established that elk were native to Nevada, but densities were likely kept low by Native American hunters wherever they occurred (9). In contrast to the CDFW Elk Management Plan, the NDOW Elk Species Management Plan noted that “Newspaper accounts report hunter kills at Lake Tahoe and Honey Lake Valley…” (10). These reports, both

near the California and Nevada border in the Sierra Nevada region, were based on an extensive search of historical newspaper and other observer records, but the specific citations were not listed. Nevertheless, these reports raise questions about the historical presence of elk in the Sierra Nevada region.

Few contemporary studies are conducted with their primary goal the determination of the historical range of a given species (11). Understanding ecosystem reference conditions in California and Nevada present challenges due to the relatively late establishment of zoological collections in museums in these states until the late nineteenth and early twentieth century (12). The California Fur Rush of the first half of the nineteenth century began as fur trappers and hunters ventured into the Sierra Nevada region beginning with Jedediah Smith in 1827 and Peter Skene Ogden in 1828 (8,13,14). These early venturers, and/or aboriginal hunters aided by the introduction of horses and firearms, may have rapidly depleted elk in the Sierra Nevada before early naturalists could document their presence. For example, after killing over 130 elk in a single winter season at the mouth of the Columbia River from November 1805 to March 1806, Lewis and Clark lamented how difficult it had become to hunt elk without traveling long distances from their camp (15). These factors may have led to similar underestimation of the historical ranges of elk and other mammalian species in California. In fact, several recent historical ecology studies have expanded the known historical range of species long thought to be non-native to the Sierra Nevada region, including physical evidence of the historical presence of North American beaver (Castor canadensis) in the high Sierra Nevada (12,16) and the historical nativity of the California red-legged frog (*Rana draytonii*) to the Yosemite Region (17). Although direct evidence of historical presence of the gray wolf (*Canis lupus*) in the Sierra

Nevada has not been found (although one museum specimen was collected near Litchfield, Lassen County, California just north of the Susan River) (18,19), wolves have recently colonized both the northern and central Sierra Nevada, with packs currently (re-)established in Plumas and Tulare Counties, respectively – suggesting that at least portions of the Sierra Nevada remains suitable wolf habitat (20). Similarly, an historical ecology study of the presence of wolverine (*Gulo gulo*) found historical observer records only in the southern subalpine and alpine Sierra Nevada (21), but recent discoveries of wolverine dispersers to the Lake Tahoe and Yosemite National Park regions in 2008 and 2023 raise questions about the former distribution of this carnivore in the Sierra Nevada (22).

The Sierra Nevada is dominated by coniferous forest, a habitat type often associated with Rocky Mountain elk, and there are no obvious biogeographical barriers that would prevent Rocky Mountain elk range expansion south from the southern Cascade Range to the northern Sierra Nevada. The uncited historical newspaper accounts mentioned above and recent studies expanding the historical range of other mammals to the Sierra Nevada, prompted our search for historical evidence of elk in the region. To overcome the paucity of evidence typical of historical ecology studies (23), we evaluated multiple different lines of evidence, similar to a recent investigation for evidence of late Holocene and historical records of bison (*Bison bison*) in northeastern California and northwestern Nevada (24). Our study goals were to research and unify broad data sources for historical and late Holocene evidence of elk in the Sierra Nevada, and to update information on the region's current (re)colonization by elk.

Methods

Study areas and time period

The Sierra Nevada mountain range is 640 km (400 miles) long on its north-south axis, and 80–130km (50–80 miles)) wide east–west. Its northern border stretches from the North Fork Feather River east to Fredonyer Pass south of Mount Lassen, then further east to the Susan River. Its southern border is at Tehachapi Pass where the Sierra Nevada give way to the Tehachapi Mountains to the southwest (12). The Sierra Nevada's peaks steadily rise in elevation from 2,130 m in the north to 4,413 m tall Mount Whitney in the south. With the exception of zooarchaeological specimens dating to the Subatlantic age (< 2,500 years BP) of the Late or Meghalayan Holocene (25), the time period of the evidence investigated to support the historic presence of elk in California beginning with the arrival of Spanish explorers in 1769 (26).

Specimen and records search

First, we searched museum records and zooarchaeological specimens for physical evidence of elk's historical presence in the Sierra Nevada region. For museum specimens we examined the Arctos Multi-Institution and Multi-Collection Museum Database (Arctos) (https://arctos.database.museum/), the Mammal Networked Information System (MaNIS) (http://manisnet.org/), and the Integrated Digitized Biocollections (iDigBio) (https://www.idigbio.org) via Boolean searches using the terms "elk", "wapiti", "Cervus canadensis", and "Cervus elaphus". In addition, we contacted curators of mammal collections at the California Academy of Sciences (CAS), the Berkeley Museum of Vertebrate Zoology (MVZ), and the National Museum of Natural History (USNM) for elk specimens collected in the Sierra Nevada region. For zooarchaeological records of elk in the region we queried FAUNMAP (http://www.ucmp.berkeley.edu/neomap/search.html) for "Cervus elaphus" and "Cervus

canadensis” remains. and references listed in recent reviews of large ungulates (bison and elk) in the Great Basin (24,27).

We used Web of Knowledge and Google Scholar to search for historical observer records by the earliest explorers and naturalists to the Sierra Nevada as well as those in citations in publications that reviewed the historical ranges of other California mammals in the region (12,19,21,28–30).

Indirect evidence search

Indirect evidence searches included searches for geographic place names using the United States Geological Survey (USGS) Geographic Names Information System (GNIS) (https://geonames.usgs.gov/pls/gnispublic) and toponomastic references for California, Nevada, and the Lake Tahoe area (31–33). Because ethnographic and ethnolinguistic information does not provide a specific written historical record, we considered it indirect evidence. We researched Native American ethnographic and ethnolinguistic information and geographic place names including the word “elk” as referenced below.

Results

Historical newspaper accounts and other observer records

First, we located the previously uncited references for the two historical newspaper accounts in the NDOW report. The first historical newspaper account was printed in The Carson Daily Appeal in 1867 (34) and reads as follows:

"ELK AT LAKE BIGLER–We learn that two elk were killed at Lake Bigler, beyond the Zephyr Cove House, during last week. One of them weighed about five hundred pounds."

- The Carson City Daily Appeal 1867.

Lake Bigler was the historical name of Lake Tahoe, named for California's third governor, John Bigler. However, because Bigler was a secessionist, the United States Department of the Interior renamed it Lake Tahoe during the Civil War (32). Zephyr Cove House was established by Andrew Gardenier, who homesteaded 65 hectares (160 acres) and built the inn in 1862 on beachfront property in Zephyr Cove, in Douglas County, Nevada on Lake Tahoe's southeastern shores, to cater to silver miners (35). The second newspaper record is of an elk hunt in the vicinity of Honey Lake Valley, Lassen County, California (36):

"RETURN OF THE HUNTERS–About two weeks since Alderman Dimock and one or two other gentlemen left this city [Virginia City] *for a hunt in the direction of Honey Lake… Night before last they returned to this city, bringing several trophies of their prowess. During their excursion they killed an elk, weighting 404 pounds, and a number of deer, antelope, and smaller game."*

- Territorial Enterprise 1868.

Honey Lake Valley is south of the Susan River, near the extreme northeastern edge of the Sierra Nevada, located 107 km north of Lake Tahoe. This account was printed in the Territorial Enterprise (Virginia City) on February 12, 1868 (Territorial Enterprise 1868). Both Honey Lake Valley and Lake Tahoe are south of the Susan River, placing both newspaper accounts in the Sierra Nevada (Fig. 2). The two newspaper accounts are 122 km and 260 km south, respectively of CDFW recognized historical elk range (Modoc County's southern border) (1).

Fig 2. Map depicting locations of nineteenth century elk hunts "H" at Honey Lake and Lake Tahoe in the Sierra Nevada. Supporting evidence includes two Late Holocene zooarchaeological records of elk "Z", and suitability of habitat reflected by presence of elk today in the northern Sierra Nevada north of Lake Tahoe and the southern Sierra Nevada about Lake Isabella.

Despite a comprehensive search, we located a single early historical observer record of elk in the Sierra Nevada region, given by Thomas Jefferson Farnham, who explored California in 1840: "*On the northwest side of this Desert* [Great Basin] *is a partially fruitful region, called the Vale of Mary's River* [Humboldt River]… *There are, however, some pretty groves of aspen and pine to be found along the stream and in the hills, among which live a few red deer and elk."* (29). Farnham also noted the occurrence of elk even further south than the Sierra Nevada in California. In 1840 he described the course of the Mojave River as it approached its headwaters in the San Bernardino Mountains, as "*gurgling through narrow vales covered with grass and fields and forests in which live the deer, the black bear, the elk, the hare, and many a singing bird*" (29). The Mojave River mainstem begins in the San Bernardino Mountains, 128 km southwest of Tehachapi Pass, the southern border of the Sierra Nevada. John Boyden "Grizzly" Adams, resided at Long Barn in the Sierra Nevada, near Sonora Pass in Tuolumne County, and described hunting elk in the 1850's but these accounts were not clearly in the mountains versus the Central Valley of California and further may have been sensationalized for the San Francisco newspapers (37).

In contrast, Jedediah Smith, the first to cross the Sierra Nevada in 1827 at Ebbett's Pass, 50 km south of Lake Tahoe, did not describe seeing elk (38). Peter Skene Ogden, the discoverer of what is now the Humboldt River in 1828 and who explored it in 1829, also did not mention elk on his return from the Humboldt Sink over the southern Cascades to Oregon (39). In the diary of Charles Preuss, who along with John Fremont were the first Anglo-Americans to see Lake Tahoe in early 1844 on Fremont's second expedition, no elk observations were reported (32).

Museum records and Late Holocene zooarchaeological specimens

There were no museum specimens found for *Cervus canadensis* or *Cervus elaphus* from the Sierra Nevada region of either California or Nevada utilizing Boolean searches of the ARCTOS, MaNIS, or iDigBio museum databases, nor did the curators of mammalogy collections we contacted have specimens that may not have been digitized or made accessible online.

Although noting that the NDOW 1997 Elk Species Management Plan did not provide publication details for the two historical newspaper accounts, it cited a comprehensive review of archaeological sites in the western Great Basin which found elk remains at two Late Holocene sites: CA-LAS-206/15 located in Secret Valley, Lassen County, California, 34 km north of Honey Lake Valley, and 26WA5649 at Dry Valley, Washoe County, Nevada, 35 km east of Honey Lake Valley (10,27) (Fig. 2).

Indirect evidence of elk in the Sierra Nevada

There are numerous place names including the word “elk” in California and Nevada, but only a single occurrence in the Sierra Nevada counties of these two states, an Elk Point in Douglas County, Nevada on Lake Tahoe (32,40). Coincidentally, Elk Point is only 0.8 km south of Zephyr Cove, although its etymology is unknown (32). Searches of other toponomastic references yielded no further elk place names in the Sierra Nevada (31,33).

In support of the historical newspaper accounts, there is ethnographic support for elk in the Sierra Nevada. The Washoe People had a word for elk, *hañakmuwe* (41). The Northern Paiute had a word for elk, but this does not provide added support since the Northern Paiute territory included portions of Oregon and Idaho where elk were historically present. However, there is a specific reference to the Honey Lake Valley Paiute, which were local to the Honey Lake Valley, stating that elk were “among the large animals captured” (42). Similarly, the Mountain Maidu’s territory likely extended at least to the western edge of the Honey Lake Valley, and although the other side of their territory extended to the edge of the Central Valley, a fairly specific reference to elk as a food item in the mountainous region states: “In the mountains, deer, elk, mountain-sheep, and bear were plenty; while in the Sacramento Valley there were great herds of antelope” (43). There is an Owens Valley Paiute legend of Hai'nanū who travels with his brother to Convict Lake then Mammoth in Mono County, California then over a ridge and shoots “soikwoi” or elk (44).

Reports of recent elk range expansions into the Sierra Nevada

The CDFW Elk Management Plan’s current elk range maps indicate that by 2017, natural range expansion of Rocky Mountain elk had occurred southwards from Modoc County through

Lassen, Plumas, and Sierra Counties, the latter 50 km north of Lake Tahoe (1). Elk calving grounds were identified in a 2020 study of the Plumas National Forest, which extends south to northern Sierra County, and reported that elk herds had been established there by the early 2000s (45). Presently, three spatially distinct herds occupy Plumas and Sierra counties, near Humbug Valley and Lake Davis, Plumas County, and Stampede Reservoir, Sierra County (CDFW internal data). Abundance and space use of these populations are not well understood, however elk occupation and reproduction in these regions indicate that habitat is suitable for elk, in at least the northern Sierra Nevada. Although the 2018 CDFW Elk Management Plan showed no elk in the southern Sierra Nevada, wildlife biologists since 2021 have observed elk, including three cows, with darker coloring consistent with Rocky Mountain elk, in the Sequoia National Forest/Lake Isabella area of Kern County at elevations above 800 m (A. Gwinn, CDFW and N. Kelly, USDA Forest Service, personal communication). These elk are thought to be dispersers from the Tehachapi herd, whose founders were Rocky Mountain elk translocated from Yellowstone National Park to the ranch of Rex Ellsworth next to Fort Tejon in Kern County’s Tehachapi Mountains in 1967 (1). These conclusions are supported by three recent documented elk-vehicle collisions on Highway 58, near Tehachapi Pass, the boundary between the Tehachapi Mountains and the southern Sierra Nevada (A. Gwinn, CDFW and N. Kelly, USDA Forest Service, personal communication). There is no evidence that the tule elk removed from Yosemite Valley in 1933 and transported to the Owens Valley in Inyo County on the southeastern edge of the Sierra Nevada (46), have expanded their range westward, as is expected due to their high fidelity for more open range.

Recent long-distance dispersals of Rocky Mountain elk in the Sierra Nevada

There are numerous recent reports of long Rocky Mountain elk dispersals southwards through the Sierra Nevada. In 2019, a radiocollared elk cow left Alturas in Modoc County and was tracked dispersing south until it was hit on U.S. 395 just north of Reno, an over 200 km linear distance (CDFW internal data). In addition, a young bull elk was filmed on a road near Weimar, Placer County in August 2022, 70 km due west of Lake Tahoe (S. Holm, CDFW, personal communication). An unusually long-distance dispersal event was detected in 2019 and 2020, when a Rocky Mountain elk was photographed on trail camera in El Dorado County near Lake Tahoe and then redetected at Saint Mary's Pass (just northwest of Sonora Pass) in Alpine County, a total of 335 km south of Modoc County and 70 km further south than Lake Tahoe, (47) (Fig. 2). Analysis of fecal DNA collected across multiple sites confirmed this individual's journey. In October 2022, a bull elk was observed by CDFW staff ~10 km south of Bridgeport in Mono County, east of US Route 395 and south of State Highway 270, a distance of 110 km south by southeast of Lake Tahoe (D. Taylor, CDFW, personal communication). In September 2023, a bull elk was observed comingling with a flock of sheep near… (D. Taylor, CDFW, personal communication). It is unknown if these observations are of distinct individuals, or the same individual detected in Alpine County in 2020.

Discussion

Multiple direct and indirect data sources indicated that elk were historically present in the northern Sierra Nevada. The two historical newspaper accounts establish the presence of elk with

reasonable specificity. A “500 pound” (227 kg) elk would not be a mule deer (*Odocoileus hemionus hemionus*), where adult bucks average 74 kg (163 lbs) dressed and about 25% heavier or 106 kg (233 lbs) live (48,49). Although mule deer size is quite variable, the largest trophy animal taken in northeastern California had a live weight of 181 kg (400 lbs) (50). The other newspaper account described a “404 pound” (184 kg) elk but differentiated it from deer and pronghorn (*Antilocapra americana*). The Honey Lake Valley newspaper account was supported by two nearby Late Holocene zooarchaeological records, one in California and one in western Nevada. This direct evidence is supported by a single reliable historical observer record near the eastern border of the Sierra Nevada by an early California explorer and naturalist, Thomas Farnham (29).

Indirect evidence also supports the historical presence of elk in the Sierra Nevada. Ethnolinguistic and ethnographic evidence from four Native American tribes place elk in the Sierra Nevada and northwestern Great Basin historically. The toponomastic reference to an Elk Point, just south of the hunting record at Zephyr Cove on the southeastern shore of Lake Tahoe, while coincidentally close to the elk hunt there, may have an eponymous or other etymology unrelated to elk, the animal. Perhaps the strongest indirect evidence for the historical presence of elk in the Sierra Nevada is habitat suitability. Habitat in the northern Sierra Nevada is clearly suitable for Rocky Mountain elk as established by steady range expansions southwards in the northern Sierra Nevada. Further expansion of the nascent Lake Isabella herd in Kern County would suggest that the southern Sierra Nevada is also suitable Rocky Mountain elk habitat.

The paucity of historical observer records and Late Holocene zooarchaeological records are consistent with elk likely occupying the Sierra Nevada at very low densities historically. An extensive review of zooarchaeological records posited that elk were never abundant in the Great Basin, finding “20 Holocene-aged Great Basin sites positive for elk, compared to 77 that contained bison (*Bison bison*)—itself never an abundant animal in this region.” (27). The lack of museum records is likely related to the relatively late establishment of museum zoology collections in California, which occur in the late nineteenth century only at the San Francisco California of Sciences and Stanford University’s Museum of Natural History–after tule elk had been nearly extirpated (12). The other factor which may drive the rarity of museum specimens and historical observer records is that the Sierra Nevada was thinly settled and poorly explored by Anglo-Americans until the latter half of the nineteenth century. Similarly, late Holocene zooarchaeological specimens may have been rare, because Late Holocene Native American populations were low, so the primary mechanism for introducing elk to caves and rock shelter archaeological sites may have occurred at low frequency (27).

Elk are vagile mammals, with reported dispersal distances up to 600 km (51). Therefore, further range expansions of Rocky Mountain elk throughout the Sierra Nevada are not only possible, but contemporary, substantiated observations suggest it is likely. Although mean dispersal distances for Rocky Mountain elk are likely 42 km or lower (51), and the above long-distance dispersals are exceptional, they do seem to be occurring more frequently in the last decade.

In conclusion, although direct evidence suggests elk were never abundant in the Sierra Nevada historically, we find that the direct and indirect evidence, when taken together, indicates that elk likely inhabited at least the northern Sierra Nevada at least as far south as Lake Tahoe. Long-distance dispersals to the central Sierra Nevada and recent colonization into its southernmost mountains, as well as a historical observer record of elk in the even more southern San Bernardino Mountains, raise the further question as to whether most of the Sierra Nevada is suitable elk habitat. Our findings should spur searches for additional physical evidence of elk in the Sierra Nevada during the historical period, utilizing novel approaches and technologies such as those that have recently expanded the recognized historical ranges of other species in California (16,52,53). Although we do not currently make recommendations for or against further elk reintroduction to the Sierra Nevada, it is clear that management activities that support and promote the recently established elk populations in Plumas and Sierra Counties may yield numerous ecological and social benefits. Sustained elk occupation in this wildfire-prone region may help in reduction of fire fuel loads (54,55), create new wildlife viewing and hunting (including indigenous hunting) opportunities (56), and reestablish an important prey base for apex predators and partial or obligate scavengers (57,58). Lastly, we recommend further research to better understand and manage the Sierra Nevada's (re)colonizing elk populations, including DNA analysis and application of satellite GPS collars to elucidate space use, habitat preferences, landscape connectivity, and identification of unoccupied habitats where range expansion by elk may naturally occur.

Acknowledgments

The authors wish to acknowledge the thoughtful diligence of Robert "Bob" McQuivey, the retired Nevada Department of Wildlife investigator who found the two historical newspaper accounts of elk in the Sierra Nevada in 1997, and whose detailed work remained unpublished for over a quarter century. We also wish to recognize Ian M. McGlory, MLIS, the Processing Archivist at Mathewson-IGT Knowledge Center, University of Nevada, Reno for his assistance locating the 1868 article in the Territorial Enterprise historical newspaper. Lastly, we thank the California Department of Fish and Wildlife biologists who provided the latest updates on the expanding elk populations in the northern and southern Sierra Nevada.

References

1. California Department of Fish and Wildlife (CDFW). Elk Conservation and Management Plan [Internet]. California Department of Fish and Wildlife; 2018 Dec p. 481. Available from: https://nrm.dfg.ca.gov/FileHandler.ashx?DocumentID=162912&inline
2. Murie OJ. The elk of North America. Harrisburg, PA, USA: Stackpole; 1951. 418 p.
3. McCullough DR. The Tule Elk: Its History, Behavior, and Ecology. Berkeley, California: Univ. of Calif. P; 1969. 209 p. (University of California publications in zoology; vol. 88).
4. Sacks BN, Lounsberry ZT, Kalani T, Meredith EP, Langner C. Development and Characterization of 15 Polymorphic Dinucleotide Microsatellite Markers for Tule Elk Using HiSeq3000. JHERED. 2016 Nov;107(7):666–9.
5. Sacks BN, Bali GK, Lounsberry ZT, Vanderzwan SL. A panel of 96 SNPs to identify individuals and differentiate subspecies of elk (Cervus canadensis) for microfluidic and other genotyping platforms. Conservation Genet Resour. 2021 Jun;13(2):111–3.
6. Merriam CH. Results of a Biological Survey of Mount Shasta, California. North American Fauna No. 16. Washington, DC, USA: United States Department of Agriculture, Division of Biological Survey; 1899. 179 p.
7. McAllister MH. Elk in Shasta County. California Fish and Game. 1919;5(2):98.
8. Dasmann RF. Environmental Changes before and after the Gold Rush. California History. 1998 1999;77(4):105–22.

9. O'Gara BW, Dundas RG. Distribution: past and present. In: North American elk: ecology and management. Washington, DC, USA: Smithsonian Institution Press; 2002. p. 67–119.

10. NDOW. Elk species management plan. [Internet]. Reno, NV, USA: Nevada Department of Wildlife (NDOW); 1997 Feb p. 66. Available from: https://www.ndow.org/wp-content/uploads/2021/10/NDOW-Elk-Species-Management-Plan-02-08-97-ADA-Compliant.pdf

11. Schwartz MK, Aubry KB, Mckelvey KS, Pilgrim KL, Copeland JP, Squires JR, et al. Inferring Geographic Isolation of Wolverines in California Using Historical DNA. J Wildl Manage. 2007;71(7):2170.

12. Lanman, R. B., Perryman, H., Dolman, B., James, C. D. The historical range of beaver in the Sierra Nevada: a review of the evidence. California Fish and Game. 2012;98(2):65–80.

13. Barbour BH. Jedediah Smith: No Ordinary Mountain Man. Norman, Oklahoma, USA: Univ of Oklahoma Press; 2011. 304 p.

14. Ogden PS. Journal of Peter Skene Ogden: Snake Expedition, 1828-1829. The Quarterly of the Oregon Historical Society. 1910 Dec;11(4):381–96.

15. Laliberte AS, Ripple WJ. Wildlife Encounters by Lewis and Clark: A Spatial Analysis of Interactions between Native Americans and Wildlife. BioScience. 2003;53(10):994.

16. James, C. D., Lanman, R. B. Novel physical evidence that beaver historically were native to the Sierra Nevada. California Fish and Game. 2012;98(2):129–32.

17. Adams AJ, Brown KC, Jennings MR, Grasso RL. Homecoming or new pad: historical evidence for California red-legged frogs and other amphibians in the Yosemite Region, California. Northwestern Naturalist. 2023 Spring;104(1):1–25.

18. Grinnell J, Dixon JS, Linsdale JM. Fur-Bearing Mammals of California. Vol. II. Berkeley, CA, USA: University of California Press; 1937. 377–777 p.

19. Schmidt RH. Gray wolves in California: their presence and absence. California Fish and Game. 1991;77(2):79–85.

20. CDFW. New Gray Wolf Pack Confirmed in Tulare County [Internet]. Sacramento, CA, USA: California Department of Fish and Wildlife; 2023 Aug [cited 2023 Aug 12]. Available from: https://wildlife.ca.gov/News/Archive/new-gray-wolf-pack-confirmed-in-tulare-county#gsc.tab=0

21. Aubry KB, Mckelvey KS, Copeland JP. Distribution and Broadscale Habitat Relations of the Wolverine in the Contiguous United States. J Wildl Manage. 2007;71(7):2147–58.

22. Moriarty KM, Zielinski WJ, Gonzales AG, Dawson TE, Boatner KM, Wilson CA, et al. Wolverine Confirmation in California after Nearly a Century: Native or Long-Distance Immigrant? Northwest Science. 2009 Apr;83(2):154–62.

23. Swetnam TW, Allen CD, Betancourt JL. Applied historical ecology: using the past to manager for the future. Ecological Applications. 1999 Nov;9(4):1189–206.

24. Grayson DK. Holocene bison in the Great Basin, western USA. The Holocene. 2006 Sep 1;16(6):913–25.

25. Walker M, Head MJ, Lowe J, Berkelhammer M, Björck S, Cheng H, et al. Subdividing the Holocene Series/Epoch: formalization of stages/ages and subseries/subepochs, and designation of GSSPs and auxiliary stratotypes. J Quaternary Science. 2019 Apr;34(3):173–86.

26. Teggart FJ. The Portola Expedition of 1769–1770. Diary of Miguel Costanso. Publications of the Academy of Pacific Coast History. 1911;2(4):162–327.

27. Grayson D, Fisher G. Holocene Elk (Cervus elaphus) in the Great Basin. In: Past, Present, and Future Issues in Great Basin Archaeology: Papers in Honor of Don D Fowler Cultural Resource Series Editor: B Hockett. Elko, NV, USA: Bureau of Land Management; 2009. p. 67–83.

28. Beidelman RG. California's frontier naturalists. Berkeley, CA, USA: University of California Press; 2006. 499 p.

29. Farnham TJ. Life, Adventures, and Travels in California. St. Louis, MO, USA: Nafis and Cornish; 1849. 413 p.

30. McCabe RE, O'Gara BW, Reeves HM. Prairie ghost: pronghorn and human interaction in early America. Boulder, CO, USA: University Press of Colorado; 2004. 175 p.

31. Gudde EG, Bright W. California place names the origin and etymology of current geographical names. 4th ed., rev.enl. Berkeley, CA, USA: University of California Press; 2010. 496 p.

32. Lekisch B. Tahoe place names: the origin and history of names in the Lake Tahoe Basin. Lafayette, Calif: Great West Books; 1988. 173 p.

33. Carlson HS. Nevada place names: a geographical dictionary. Reno, NV, USA: University of Nevada Press; 1985. 296 p.

34. Carson Daily Appeal. Elk at Lake Bigler. Carson Daily Appeal. 1867 Oct 17;3.

35. Cahill N. On the road: This iconic High Sierra Lake was once named…Bigler? Western Water (Water Education Foundation) [Internet]. 2018 May 4; Available from:

https://www.watereducation.org/western-water/road-iconic-high-sierra-lake-was-once-namedbigler

36. Territorial Enterprise. Return of the hunters. Territorial Enterprise. 1868 Feb 12;2.

37. Hittell TH. The Adventures of James Capen Adams: Mountaineer and Grizzly Bear Hunter, of California. San Francisco, CA, USA: Towne & Bacon; 1860. 378 p.

38. Smith AJ. Men against the mountains.;: Jedediah Smith and the South West Expedition of 1826-1829. New York, NY, USA: John Day Company; 1965. 320 p.

39. Scaglione J, Ogden PS. Ogden's Report of His 1829-1830 Expedition. California Historical Society Quarterly. 1949 Jun 1;28(2):117–24.

40. U.S. Geological Survey. GNIS: geographic names information system: digital gazetteer [Internet]. Reston, VA, USA: U.S. Geological Survey, National Mapping Division; Available from: https://edits.nationalmap.gov/apps/gaz-domestic/public/search/names

41. Kroeber AL. The Washo Language of East Central California and Nevada. University of California Publications in American Archaeology and Anthropology. 1907 Sep;4(6):251–317.

42. Riddell FA. Honey Lake Paiute ethnography. Nevada State Museum Anthropological Papers. 1960 Dec;4:137.

43. Dixon RB. The Huntington California Expedition. The Northern Maidu. Bulletin of the American Museum of Natural History. 1905 May;XVII(III):119–346.

44. Steward JH. Myths of the Owens Valley Paiute. University of California Publications in American Archaeology and Ethnology. 1936;34(5):355–440.

45. Marshall A. Investigating calving areas for Rocky Mountain elk on the Plumas National Forest: Implications for land management. Thesis [Internet]. [Corvallis, OR, USA]: Oregon State University; 2020. Available from: https://ir.library.oregonstate.edu/downloads/dv140129z

46. Moffitt J. History of the Yosemite Elk Herd. California Fish and Game. 1934 Jan;20(1):37–51.

47. CDFW. Following the unusual migration of a trailblazing elk. [Internet]. Sacramento, CA, USA: California Department of Fish and Wildlife (CDFW); 2020 Jun [cited 2023 Jun 6]. (CDFW Science Spotlight). Available from: https://wildlife.ca.gov/Science-Institute/News/following-the-unusual-migration-of-a-trailblazing-elk

48. Heffelfinger J, Krausman PR, Cox A, editors. Ecology and management of black-tailed and mule deer of North America. First edition. Boca Raton, FL: CRC Press; 2023. 536 p.

49. Mackie RJ, Kie JG, Pac DF, Hamlin KL. Mule Deer (Odocoileus hemionus). In: Wild Mammals of North America: Biology, Management, and Conservation. 2nd ed. Baltimore, MD, USA: The Johns Hopkins University Press; 2003. p. 889–905.

50. Dasmann WP. Big Game of California. Sacramento, CA, USA: California Fish and Game; 1975. 57 p.

51. Yott A, Rosatte R, Schaefer JA, Hamr J, Fryxell J. Movement and Spread of a Founding Population of Reintroduced Elk (Cervus elaphus) in Ontario, Canada. Restoration Ecology. 2011 Jan;19(101):70–7.

52. Chase, Shawn D., Manning, David J., Cook, David G., White, Sean K. Historic Accounts, Recent Abundance, and Current Distribution of Threatened Chinook Salmon in the Russian River, California. California Fish and Game. 2007 Jun;93(3):130–48.

53. Lanman RB, Hylkema L, Boone CM, Allée B, Castillo RO, Moreno SA, et al. Ancient DNA analysis of archaeological specimens extends Chinook salmon's known historic range to San Francisco Bay's tributaries and southernmost watershed. PLoS One. 2021;16(4):e0244470. doi: 10.1371/journal.pone.0244470

54. Lecomte X, Caldeira MC, Catry FX, Fernandes PM, Jackson RB, Bugalho MN. Ungulates mediate trade-offs between carbon storage and wildfire hazard in Mediterranean oak woodlands. Mukul S, editor. J Appl Ecol. 2019 Mar;56(3):699–710.

55. Defrees DH, Averett JP, Wisdom MJ, Endress BA. Interactive effects of fuels reduction and large herbivores on shrub assemblages in dry conifer forests of the interior west, USA. Forest Ecology and Management. 2020 May;463:118031.

56. USFWS. 2011 National survey of fishing, hunting, and wildlife-associated recreation State overview. [Internet]. Washington, DC, USA: U.S. Fish and Wildlife Service; 2012. Available from: https://digitalmedia.fws.gov/digital/collection/document/id/858/

57. Emslie SD. Age and diet of fossil California condors in Grand Canyon, Arizona. Science. 1987 Aug 14;237(4816):768–70.

58. Grubb TG, Lopez RG. Winter Scavenging of Ungulate Carrion By Bald Eagles, Common Ravens, and Coyotes In Northern Arizona. Journal of Raptor Research. 2018 Dec;52(4):471–83.

Figure 1

Click here to access/download;Figure;Figure 1 CDFW Historic Elk Range.tiff

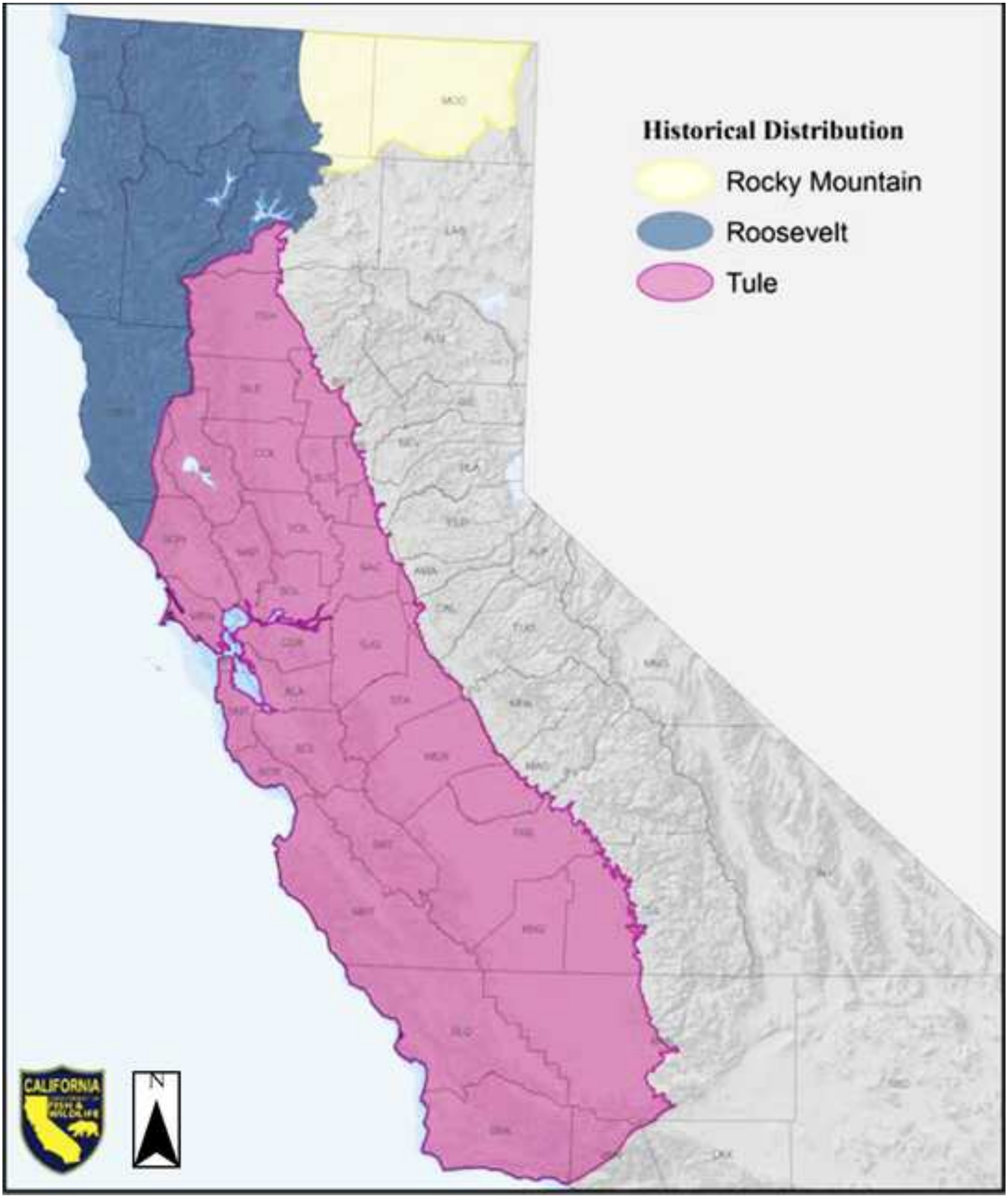

Figure 2

Click here to access/download;Figure;Figure 2 Evidence Elk in Sierra Nevada.tiff

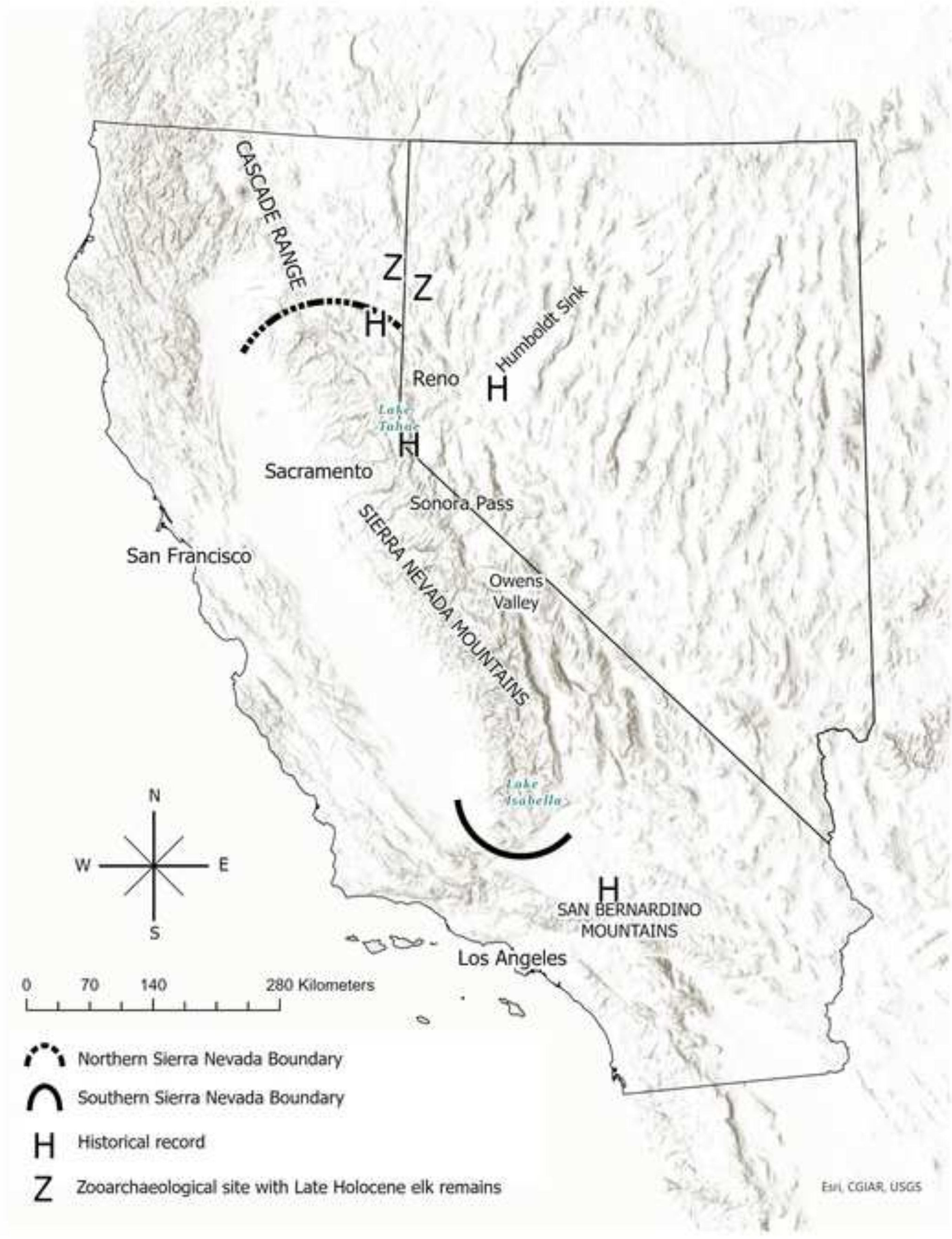

Supplement: Attachment — Submitted filename: PONE-D-23-41818 CS review.pdf [file pone.0301665.s001.pdf]
