# Supplementary material for: Novel evidence that elk were historically native to the Sierra Nevada, and recent range expansions into the region
Source: PLoS One. 2024 Sep 18;19(9):e0301665. doi: 10.1371/journal.pone.0301665 (PMC11410259; doi:10.1371/journal.pone.0301665)
Supplement: Attachment — Submitted filename: Response to Reviewers 2024-02-18.docx [file pone.0301665.s002.docx]

**Response to Reviewers for Manuscript Revision** - **Novel evidence that elk were historically native to the Sierra Nevada, and recent range expansions into the region**

| **Editor Comments** | **Authors Response (line numbers given below are for the Revised Manuscript with Track Changes)** |
| --- | --- |
| After careful consideration, we feel that it has merit but does not fully meet PLOS ONE’s publication criteria as it currently stands. Therefore, we invite you to submit a revised version of the manuscript that addresses the points raised during the review process. | Thank you, the editor and reviewer comments are helpful and certainly addressable. Our responses are below. |
| 1. LInes 26 and again on lines 66 and 67: Not much evidence presented in the paper to substantiate the claim of elk populations being suppressed by aboriginal hunting . I would think even if native american hunted elk there would be evidence from petroglyphs, or bone fragments in archeology sites. Please address. | On review, we agree, and have deleted the two mentions of suppression of elk numbers by aboriginal hunting in the Abstract line 39 and the Introduction line 78 of the Revised Manuscript with Track Changes. |
| 2. Lines 190-193: Although the authors say this is in the Sierra Nevada region. The reference seems to be more relegated to Central to Northeast NV, where the Humboldt River begins (Marys River) and terminates (Humboldt Sink) near Lovelock, NV. I don't know that this provides direct or indirect evidence but it does indicate elk may have been present in the region around the 1840's. Please clarify or modify this claim. | Again we agree, having reviewed the eastern border of the Sierra Nevada, which although it extends east into Nevada, the Humboldt Sink is beyond it - in the Great Basin. Although the Sierra Nevada includes the Zephyr Cove, Nevada historical record, and the Sierra Nevada extends further east into Nevada, it is merely adjacent to the terminus of the Humboldt River. Accordingly, we have changed lines 224-225 to indicate there were no additional records in the Sierra Nevada, but one in the adjacent Great Basin’s Humboldt Sink. Ditto on lines 361-363. |
| 3. Lines 198-203: Same general comment as above. Consider modifying the description here and mentioning in the Discussion. | Fig 2 caption line 214 now explicitly states that the zooarchaeological records of elk “Z” are located in the extreme northwest Great Basin, just north of the Sierra Nevada. Also, we modified the Discussion lines 352-355 in two ways: to clarify that the two zooarchaeological specimens were outside of the Sierra Nevada but adjacent to it in the northwest Great Basin. Also we clarified that both of Farnham’s historical observer records were outside the Sierra Nevada but east and south of it, respectively. lines 224-225 |
| 4. Lines 280-284: It is not clear where this observation is noted on Figure 2. | We added Contemporary Observer Records (C) to Fig. 2 for St. Mary’s Pass to make the location clear, and also for Lake Isabella in the south and Stampede Reservoir in the north to show how far herds have now colonized/recolonized the Sierra Nevada. |
| 5. Lines 289-290: I'm not sure if this is a reference to all the observations from 2019 to 2022, or just the observations that were corroborated with fecal DNA? Were they at least from different sexes of elk? That would lend more plausibility of there being a breeding population in the Sierra Nevada. Please clarify | We have clarified that fecal DNA collected near Lake Tahoe, Saint Mary’s Pass, and intervening sites confirmed this journey was made by the same individual elk – lines 335-336. We also clarified that breeding populations have colonized three locations including Stampede Reservoir in the north now made clearer by addition of “three spatially distinct herds composed of both sexes and varying age classes occupy Plumas and Sierra counties, near Humbug Valley and Lake Davis, Plumas County, and Stampede Reservoir, Sierra County” on lines 296-298. |
| Minor critique: some language could be tightened throughout. For example, line 22. Consider rewording to be more concise: "were previously considered non-native to the Sierra Nevada" perhaps.3` | Thank you, change made with tighter language in line 22, as well as lines 44-45, and many other spots. |
| **Note to Editor and Reviewers** | While in review, a highly relevant article by Sacks et al. 2024 in the Journal of Wildlife Management was published – it is new reference 49. This genetic study of 1,204 California elk individuals found that the elk moving into the northern Sierra Nevada are hybrid Roosevelt and Rocky Mountain elk. This is now referenced on line 299-300 and 417, and we added to the Discussion that because of this genetic admixture, the “hybrid may be better adapted to higher elevations and novel terrain than their non-hybridized ancestors”. lines 417-418 |
| **Reviewer #1 Comments:** |  |
| 1. I believe this manuscript is an important contribution to science and understanding of the history of elk distribution in California and in the Sierra Nevada mountains and surrounding areas. The manuscript is well written and makes a compelling argument that Rocky Mountain elk likely inhabited the Sierra Nevada previously to the 1900's, but due to a lack of museum specimens, zooarchaeological or other records, previous texts and publications may have overlooked their presence. This could have been due to a lack of modern records documenting their existence because they were overexploited or simply vary rare, albeit present, in modern history. I believe the authors present more indirect evidence of this than direct evidence. The historical records could be substantiated by more clear references than were presented here. | Thank you, and we believe that we have clarified the several references to locations as suggested by the Editor above. We also added in the Discussion lines 371 that indirect evidentiary records “were more numerous than direct evidentiary records.” |
| 2. Furthermore, the observations and zooarchaeological records that are presented as "direct evidence" seem to be more indirect or circumstantial to elk being near the Sierra Nevada mountains or perhaps occasional dispersals to the Sierra Nevada. For example the map on Figure 2 shows the locations of historical records of elk and zooarchaeological sites with elk remains, however only 2 out of 6 records appear to be within the identified Sierra Nevada zone while the other 4 records occur just outside of this zone. | We agree, and have noted that while the two historical elk hunts were within the Sierra Nevada, that the two zooarcheological records were outside it in the Great Basin, albeit adjacent. Similarly, the two historical observer records by Farnham were outside and better described as east and south of the Sierra Nevada, respectively – as noted above in response to the Editor’s comment. |
| 3. The indirect evidence seems more compelling including the ethnographic and ethnolinguistic information presented such as the newspaper articles describing elk being harvested in the Sierra Nevada as well as local place names and native American names for elk in the region and specifically within the Sierra Nevada. | As in 1. we have added emphasis that the indirect records were markedly more numerous than the direct ones. Also the 1^st^ sentence in the last Conclusion paragraph makes this point: “In conclusion, although direct evidence suggests elk were never abundant in the Sierra Nevada historically, we find that the direct and indirect evidence, when taken together, indicates that elk likely occupied the northern Sierra Nevada at least as far south as Lake Tahoe, as well as the northwest Great Basin, at least seasonally, in low densities, or both.” lines 456-459 |
| 4. Additionally, as the title implies there has been a recent influx and establishment of Rocky Mountain elk into portions of the northern Sierra Nevada and several documented recent sightings or telemetry movements elsewhere into the Sierra Nevada indicating it is clearly suitable habitat for them. This seems relevant to modern management of both these novel ungulates and the predator prey relationships they may arise from their expansion. This may be very important for understanding the distribution of other species such as modern Gray wolves that have established and also other large carnivores who prey on these species such as mountain lions, black bears, coyotes, and possibly wolverines that scavenge these carcasses. I believe this provides some more evidence that elk could potentially have occurred in the Sierra Nevada historically, assuming there has not been major habitat changes in the past 150 years, and that they likely can persist there in current conditions. | This is an excellent point and so we have added: The recent recolonization of elk’s primary predator, gray wolves, in Tulare County, California in the southern Sierra Nevada, may also imply that elk are a missing component of the ecosystem. to lines 420-422. |
| 5. Overall I would recommend publishing this manuscript with a few slight clarifications to references provided and some minor editorial suggestions. | Thank you for your helpful comments and we hope that the additions now make the manuscript satisfactory. |
| **Journal Requirements** |  |
| 1. Please ensure that your manuscript meets PLOS ONE's style requirements, including those for file naming. | Fixed references to proper formatting, especially of references. |
| 2. Add whether permits were required. | Added lines 173-174: Contemporary observer records were obtained by CDFW and NDOW biologists. No permits were required for the described study, which complied with all relevant regulations. |
| 3. We note that Figures 1 & 2 in your submission contain [map/satellite] images which may be copyrighted. | For Fig. 1 we obtained permission from CDFW and added to the Figure caption: Reprinted from CDFW Elk Conservation and Management Plan under a CC BY license, with permission from CDFW, original copyright 2018. lines 72-74  For Fig. 2 we added to the Figure caption that the images were collected from public domain sources, as noted in bottom right of the figure as “Esri, CGIAR, USGS”. lines 220-221 |
